# Robust Sparse Underwater Acoustic Channel Estimation Using a Bidirectional Proportionate Recursive Maximum Correntropy Criterion Algorithm

**DOI:** 10.3390/e28070786

**Published:** 2026-07-12

**Authors:** Xiao-Chen Chen, Guan-Quan Dai, Yang Shi, Fei-Yun Wu

**Affiliations:** 1 College of Navigation, Jimei University, Xiamen 361021, China; 202411823026@jmu.edu.cn (X.-C.C.); 14018@qztc.edu.cn (G.-Q.D.); 202512861046@jmu.edu.cn (Y.S.); 2 College of Transportation and Navigation, Quanzhou Normal University, Quanzhou 362000, China

**Keywords:** underwater acoustic communication, sparse channel estimation, bidirectional filtering, maximum correntropy criterion, proportionate update, non-Gaussian noise, block-based channel tracking

## Abstract

Aiming at the problem that sparse channel estimation in underwater acoustic communication is susceptible to complex multipath propagation, non-Gaussian impulsive noise, and channel time variations, this paper proposes a bidirectional proportionate recursive maximum correntropy criterion algorithm, referred to as Bi-PRMCC. By introducing a bidirectional filtering structure into the proportionate recursive maximum correntropy criterion (PRMCC) framework, the proposed algorithm jointly exploits the information from forward and backward data sequences, thereby improving the estimation accuracy and block-based channel variation tracking capability for sparse underwater acoustic channels. Meanwhile, the maximum correntropy criterion enhances the robustness of the algorithm against non-Gaussian impulsive noise and outlier error samples, while the proportionate update mechanism improves its identification capability for dominant taps in sparse channels. To verify the effectiveness of the proposed algorithm, short-range sparse underwater acoustic channels and long-range complex multipath underwater acoustic channels are constructed based on the Bellhop ray-tracing model. Simulation experiments are then conducted under three typical non-Gaussian noise environments, namely Cauchy noise, α-stable distribution noise, and Middleton noise. The experimental results show that, compared with recursive least squares (RLS), bidirectional recursive least squares (Bi-RLS), proportionate recursive least squares (PRLS), recursive maximum correntropy criterion (RMCC), and PRMCC, Bi-PRMCC achieves a lower steady-state normalized mean square deviation (NMSD) under different non-Gaussian noise conditions, indicating stronger robustness against impulsive noise. Under different signal-to-noise ratio conditions, the proposed algorithm still maintains superior steady-state estimation performance. In addition, in the channel abrupt-change tracking experiment, Bi-PRMCC can rapidly reconverge after channel variations occur, demonstrating favorable reconvergence capability under abrupt channel variations. The ablation study further verifies the stable performance gain brought by the bidirectional structure to PRMCC. Overall, the proposed Bi-PRMCC algorithm exhibits high estimation accuracy, robustness, and reconvergence capability under complex non-Gaussian noise and abrupt channel variation conditions.

## 1. Introduction

Underwater acoustic (UWA) communication is essential for ocean observation, underwater vehicles, and marine resource exploration. However, UWA channels suffer from limited bandwidth, long delay spread, severe multipath propagation, and strong time variation, which make reliable transmission and channel estimation challenging. In shallow water, surface and bottom reflections further intensify inter-symbol interference and estimation errors. Therefore, accurate and robust adaptive channel estimation [1] is critical for high-performance UWA communication.

Practical UWA channels are usually sparse [2], since most energy is concentrated on a few dominant propagation paths while many taps are nearly zero. To exploit this property, sparse adaptive filtering algorithms [3,4] have been widely studied. Although recursive least squares (RLS) [5] converges rapidly and has good tracking ability, it does not sufficiently exploit channel sparsity, which limits its performance in sparse multipath environments.

To improve sparse channel estimation, proportionate updating mechanisms [6], sparse norm constraints [7], and zero-attracting strategies have been introduced into adaptive filtering. These methods assign larger update gains to dominant taps and suppress zero or near-zero coefficients, thereby improving sparse channel identification accuracy [8]. In UWA applications, $l_{1}$-PRLS [9], $l_{0}$-PRLS, and related proportionate sparse algorithms have shown better performance than conventional RLS. However, most of them are still based on the mean square error criterion and are sensitive to large-amplitude outliers under impulsive noise [10,11].

In realistic marine environments, ambient noise often deviates from the Gaussian assumption because of biological activity, ship noise, mechanical vibration, and burst interference. Under such non-Gaussian conditions, algorithms based on second-order statistics and the minimum mean square error criterion [12,13,14] may suffer from distorted weight updates and degraded steady-state accuracy. To enhance robustness, kernel-based criteria have been introduced into adaptive filtering. Among them, the maximum correntropy criterion (MCC) [15] down-weights large error samples through a Gaussian kernel and thus can effectively suppress impulsive outliers [16].

Based on MCC, the recursive maximum correntropy criterion (RMCC) algorithm [17] combines robust error measurement with recursive estimation and achieves improved performance under non-Gaussian noise. The proportionate recursive maximum correntropy criterion (PRMCC) algorithm [18] further introduces proportionate updating into RMCC, enabling the estimator to exploit sparse channel structures while maintaining robustness against impulsive noise. Other robust criteria have also been studied for sparse system identification. For example, kernel risk-sensitive loss performs well in impulsive noise [19], and its proportionate and sparsity-constrained variants [20] further show that combining robust criteria with sparse-aware updating [21,22] can improve convergence and reduce steady-state error.

Despite these advances, most kernel-based sparse adaptive filtering algorithms still use a unidirectional recursive structure, where estimation is performed only along the forward time direction. Thus, useful information contained in the reverse training sequence is not fully utilized [23]. Recently, bidirectional processing has attracted attention in UWA equalization and channel estimation because it can jointly use forward and backward data sequences [24]. Existing bidirectional equalizers and bidirectional joint iterative $l_{1}$-RLS methods [25] have demonstrated the benefit of combining sparsity and sequence-direction diversity. However, the integration of bidirectional processing with MCC-based robust criteria [26,27] and proportionate sparse updating remains insufficiently explored.

Although sparse constraints, robust kernel criteria, and bidirectional structures have individually improved UWA channel estimation, several limitations still remain. Sparse RLS-type algorithms [28] are effective for sparse channels but are vulnerable to impulsive outliers, while RMCC and PRMCC improve robustness but mainly rely on unidirectional recursion. Therefore, a unified algorithm that jointly exploits sparse prior information [29], robust error measurement, and bidirectional sequence information is still needed.

Motivated by the above analysis, this paper proposes a bidirectional proportionate recursive maximum correntropy criterion algorithm, termed Bi-PRMCC, for sparse UWA channel estimation [30] under non-Gaussian noise. The proposed method introduces a forward–backward filtering structure into PRMCC and fuses the two estimation results through weighted combination. In this way, proportionate updating enhances dominant-tap identification, MCC suppresses impulsive outliers, and bidirectional filtering improves training-sequence utilization.

To evaluate the proposed algorithm, two representative shallow-water acoustic channels are constructed using the Bellhop ray-tracing model, including a short-range sparse channel and a long-range complex multipath channel. Bi-PRMCC is compared with RLS, Bi-RLS, PRLS, RMCC, and PRMCC under Cauchy noise, α-stable noise, Middleton noise, different SNR levels, and abrupt channel variations. Simulation results show that Bi-PRMCC achieves lower steady-state NMSD, stronger robustness against impulsive interference, and better reconvergence capability after channel changes. Ablation studies further verify that the bidirectional structure brings stable performance gains when incorporated into PRMCC.

The main contributions of this paper are summarized as follows.

First, a bidirectional PRMCC framework is formulated for sparse UWA channel estimation under non-Gaussian impulsive noise. Different from a direct use of PRMCC in a single time direction, the proposed Bi-PRMCC constructs both forward and reverse recursive estimators under the maximum correntropy criterion and proportionate adaptation mechanism. Therefore, robust error suppression and sparse-tap enhancement are performed in both data directions rather than being added as separate post-processing operations.

Second, a weighted forward–backward fusion mechanism is introduced to combine the two recursive estimates. This mechanism enables the algorithm to exploit complementary information from the forward and reverse training sequences. The influence of the fusion weights is further analyzed, and the balanced setting $\eta_{f}=\eta_{r}=0.5$ is shown to provide the best overall steady-state performance in the considered scenario.

Third, a segment-wise reverse-indexing strategy is adopted for abrupt channel-change conditions. This strategy restricts the backward recursion within the same channel-state segment, avoiding the mixing of samples belonging to different channel states. Hence, the proposed method is more suitable for block-based sparse channel estimation under channel variations.

Finally, comprehensive simulations, including non-Gaussian noise tests, SNR analysis, abrupt channel-change experiments, ablation studies, parameter sensitivity analysis, running time comparison, and measured UWA channel validation, are conducted to verify that the performance improvement of Bi-PRMCC comes from the joint effect of correntropy-based robustness, proportionate sparse adaptation, and bidirectional information fusion.

The remainder of this paper is organized as follows. Section 2 introduces the sparse UWA channel estimation model. Section 3 presents the principle, derivation, and implementation of Bi-PRMCC. Section 4 evaluates the proposed algorithm under different noise models, SNR levels, abrupt channel changes, and ablation settings. Finally, Section 5 concludes this paper.

## 2. Sparse Underwater Acoustic Channel Estimation Model

In an underwater acoustic communication system, the transmitted signal usually reaches the receiver through multiple propagation paths caused by sea-surface reflection, seabed reflection, and inhomogeneity of the water medium. Since only a small number of propagation paths usually have dominant energy, the discrete underwater acoustic channel can be modeled as a finite-length sparse channel impulse response.

Let the transmitted training sequence be s(n) and the channel length be *L*. The input vector at time instant *n* is defined as

(1) $$x\left(n\right)={\left[s\left(n\right),s\left(n-1\right),\ldots ,s\left(n-L+1\right)\right]}^{T}.$$

For the complex baseband signal model, the received signal is expressed as

(2) $$d\left(n\right)=h_{0}^{H}x\left(n\right)+v\left(n\right),$$

where d(n) denotes the received signal, x(n) is the input vector constructed from the known training sequence, $h_{0}$ is the unknown equivalent channel vector to be estimated, v(n) is the additive noise, and ${\left(\cdot \right)}^{H}$ denotes the conjugate transpose.

For sparse underwater acoustic channels, most elements of $h_{0}$ are zero or close to zero, and only a few taps corresponding to dominant propagation paths have significant amplitudes. Therefore, the sparsity of the channel can be described as

(3) $$∥h_{0}{∥}_{0}=K,\;K\ll L,$$

where *K* denotes the number of significant channel taps. This sparse structure implies that the main channel energy is concentrated on a small number of dominant taps.

Let $\hat{h}\left(n\right)$ denote the channel estimate at the *n*th iteration. Before updating the filter coefficients, the predicted output is given by

(4) $$\hat{y}\left(n\right)={\hat{h}}^{H}\left(n-1\right)x\left(n\right).$$

The corresponding estimation error is defined as

(5) $$e\left(n\right)=d\left(n\right)-\hat{y}\left(n\right).$$

The objective of sparse channel estimation is to estimate the unknown sparse channel vector $h_{0}$ from the known input vector x(n) and the received signal d(n). In proportionate adaptive filtering algorithms, different step sizes or updating gains are assigned to different channel taps according to their relative magnitudes. As a result, the dominant taps can receive larger updating gains, whereas the insignificant taps are assigned smaller gains. In this way, the sparse structure of the underwater acoustic channel can be exploited without explicitly introducing an $\ell_{1}$-norm regularization term.

During the iterative process, the adaptive algorithm updates the channel estimate $\hat{h}\left(n\right)$ according to the error signal e(n) and the proportionate gain mechanism. By allocating larger adaptation gains to significant taps and smaller gains to insignificant taps, the algorithm aims to improve the convergence speed and estimation accuracy for sparse underwater acoustic channels. As the iteration proceeds, the estimated channel vector $\hat{h}\left(n\right)$ gradually approaches the true sparse channel vector $h_{0}$.

## 3. Proposed Bi-PRMCC Algorithm

Based on the channel estimation model described above, this paper proposes a bidirectional proportionate recursive maximum correntropy criterion algorithm, namely Bi-PRMCC. The proposed algorithm takes PRMCC as the basic recursive update unit and introduces a bidirectional iterative mechanism. By simultaneously exploiting the forward and backward information of the training data, the steady-state accuracy and tracking capability of sparse underwater acoustic channel estimation can be improved.

### 3.1. Basic Recursive Process of PRMCC

Let the input vector, received signal, and estimated channel vector at the *n*th iteration be denoted by x(n), d(n), and $\hat{h}\left(n\right)$, respectively. At the beginning of the *n*th iteration, based on the estimate obtained at the previous time instant, $\hat{h}\left(n-1\right)$, the predicted output of the filter is given by

(6) $$\hat{y}\left(n\right)={\hat{h}}^{H}\left(n-1\right)x\left(n\right).$$

The corresponding estimation error is expressed as

(7) $$e\left(n\right)=d\left(n\right)-\hat{y}\left(n\right),$$

where the superscript *H* denotes the conjugate transpose. To improve the robustness of the algorithm in non-Gaussian impulsive noise environments, PRMCC adopts the maximum correntropy criterion to weight the error. The corresponding error weighting factor is defined as

(8) $$\phi \left(n\right)=\exp\left(-\frac{{\left|e\left(n\right)\right|}^{2}}{2\sigma^{2}}\right),$$

where σ is the kernel width parameter. When the error magnitude is large, the value of ϕ(n) decreases significantly, thereby reducing the influence of abnormal error samples on the filter update.

The corresponding gain vector is given by

(9) k(n)=P(n−1)x(n)λ+ϕ(n)RexH(n)P(n−1)x(n),

where P(n) denotes the inverse correlation matrix, λ is the forgetting factor, and Re{·} denotes the real-part operator. The inverse correlation matrix is then updated as

(10) $$P\left(n\right)=\lambda^{-1}\left[P\left(n-1\right)-\phi \left(n\right)k\left(n\right)x^{H}\left(n\right)P\left(n-1\right)\right].$$

To exploit the sparsity of underwater acoustic channels, PRMCC introduces a proportionate matrix G(n−1) into the weight update. This matrix is a diagonal matrix defined as

(11) $$G\left(n-1\right)=\mathrm{diag}\left\{g_{1}\left(n-1\right),g_{2}\left(n-1\right),\ldots ,g_{L}\left(n-1\right)\right\}.$$

The *l*th diagonal element is given by

(12) $$g_{l}\left(n-1\right)=\theta \left[\frac{1-\alpha }{2L}+\frac{\left(1+\alpha \right)|{\hat{h}}_{l}\left(n-1\right)|}{2{∥\hat{h}\left(n-1\right)∥}_{1}+\varepsilon }\right],$$

where *L* is the channel length, α is the proportionate control parameter, θ is the trace control parameter of the proportionate matrix, and ε is a small positive constant used to avoid division by zero.

Therefore, the channel estimation update formula of PRMCC is expressed as

(13) $$\hat{h}\left(n\right)=\hat{h}\left(n-1\right)+G\left(n-1\right)k\left(n\right)\phi \left(n\right)e^{*}\left(n\right),$$

where $e^{*}\left(n\right)$ denotes the complex conjugate of the error. Through the proportionate matrix G(n−1), dominant channel taps with larger amplitudes can obtain larger update weights, whereas non-dominant taps with smaller amplitudes are updated with relatively smaller steps. Consequently, the estimation capability of the algorithm for sparse underwater acoustic channels is improved.

### 3.2. Bidirectional PRMCC Iterative Structure

Although PRMCC can combine the maximum correntropy criterion with the proportionate update mechanism to improve sparse channel estimation performance under non-Gaussian noise, its recursive process remains unidirectional. To further utilize the effective information contained in the training data, this paper introduces a bidirectional iterative mechanism into PRMCC and constructs the Bi-PRMCC algorithm.

Bi-PRMCC consists of two parallel recursive branches: a forward PRMCC branch and a backward PRMCC branch. The forward branch performs recursive estimation according to the original data order, while the backward branch performs recursive estimation using the data in reverse order. The two branches obtain the forward and backward channel estimates, respectively, which are then fused through weighted combination to generate the final estimation result.

It should be noted that the proposed bidirectional recursion is implemented in a block-based or segment-wise manner. Since the backward branch uses the samples in reverse order within the current data segment, the whole segment should be available before the backward recursion is performed. Therefore, Bi-PRMCC is not claimed as a strictly sample-by-sample zero-delay online estimator. In practical implementation, a buffer is required to store one data segment, and the corresponding processing latency is related to the segment length. If the length of the current segment is denoted by $N_{s}$, the required buffer length is $N_{s}$ samples, and the worst-case delay is approximately one segment duration.

**Figure 1:**
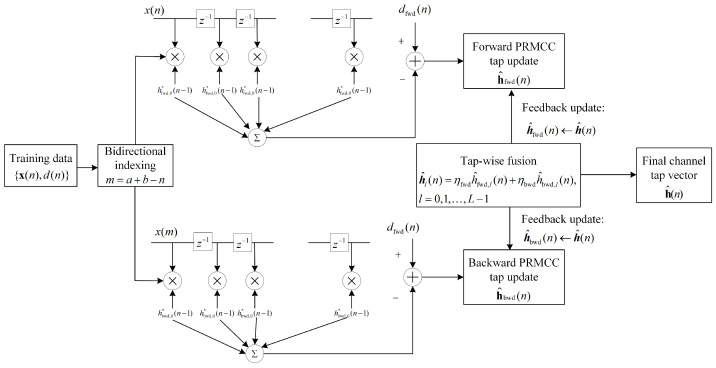
Bidirectional iterative channel estimation framework of the Bi-PRMCC algorithm.

The bidirectional iterative channel estimation framework of Bi-PRMCC is shown in Figure 1.

In the forward branch, the input vector and received signal are denoted by $x_{\mathrm{fwd}}\left(n\right)$ and $d_{\mathrm{fwd}}\left(n\right)$, respectively, and the estimated channel vector is denoted by ${\hat{h}}_{\mathrm{fwd}}\left(n\right)$. The prediction error is given by

(14) $$e_{\mathrm{fwd}}\left(n\right)=d_{\mathrm{fwd}}\left(n\right)-{\hat{h}}_{\mathrm{fwd}}^{H}\left(n-1\right)x_{\mathrm{fwd}}\left(n\right).$$

The corresponding maximum correntropy error weighting factor is

(15) $$\phi_{\mathrm{fwd}}\left(n\right)=\exp\left(-\frac{|e_{\mathrm{fwd}}{\left(n\right)|}^{2}}{2\sigma^{2}}\right).$$

The forward gain vector is calculated as

(16) kfwd(n)=Pfwd(n−1)xfwd(n)λ+ϕfwd(n)RexfwdH(n)Pfwd(n−1)xfwd(n).

The forward proportionate matrix $G_{\mathrm{fwd}}\left(n-1\right)$ is computed according to ${\hat{h}}_{\mathrm{fwd}}\left(n-1\right)$. Then, the forward channel estimate is updated by

(17) $${\hat{h}}_{\mathrm{fwd}}\left(n\right)={\hat{h}}_{\mathrm{fwd}}\left(n-1\right)+G_{\mathrm{fwd}}\left(n-1\right)k_{\mathrm{fwd}}\left(n\right)\phi_{\mathrm{fwd}}\left(n\right)e_{\mathrm{fwd}}^{*}\left(n\right),$$

and the forward inverse correlation matrix is updated as

(18) $$P_{\mathrm{fwd}}\left(n\right)=\lambda^{-1}\left[P_{\mathrm{fwd}}\left(n-1\right)-\phi_{\mathrm{fwd}}\left(n\right)k_{\mathrm{fwd}}\left(n\right)x_{\mathrm{fwd}}^{H}\left(n\right)P_{\mathrm{fwd}}\left(n-1\right)\right].$$

In the backward branch, the algorithm does not simply duplicate the forward branch. Instead, it selects samples recursively according to the reverse index within the current data segment. Let the start and end positions of the channel segment containing the current iteration *n* be denoted by *a* and *b*, respectively. The backward sample index is defined as

(19) m(n)=a+b−n.

The corresponding backward input vector and received signal are denoted by $x_{\mathrm{bwd}}\left(n\right)$ and $d_{\mathrm{bwd}}\left(n\right)$, respectively. The prediction error of the backward branch is given by

(20) $$e_{\mathrm{bwd}}\left(n\right)=d_{\mathrm{bwd}}\left(n\right)-{\hat{h}}_{\mathrm{bwd}}^{H}\left(n-1\right)x_{\mathrm{bwd}}\left(n\right).$$

The corresponding error weighting factor is

(21) $$\phi_{\mathrm{bwd}}\left(n\right)=\exp\left(-\frac{|e_{\mathrm{bwd}}{\left(n\right)|}^{2}}{2\sigma^{2}}\right).$$

The backward gain vector is calculated as

(22) kbwd(n)=Pbwd(n−1)xbwd(n)λ+ϕbwd(n)RexbwdH(n)Pbwd(n−1)xbwd(n).

The backward proportionate matrix $G_{\mathrm{bwd}}\left(n-1\right)$ is computed according to ${\hat{h}}_{\mathrm{bwd}}\left(n-1\right)$. Then, the backward channel estimate is updated by

(23) $${\hat{h}}_{\mathrm{bwd}}\left(n\right)={\hat{h}}_{\mathrm{bwd}}\left(n-1\right)+G_{\mathrm{bwd}}\left(n-1\right)k_{\mathrm{bwd}}\left(n\right)\phi_{\mathrm{bwd}}\left(n\right)e_{\mathrm{bwd}}^{*}\left(n\right),$$

and the backward inverse correlation matrix is updated as

(24) $$P_{\mathrm{bwd}}\left(n\right)=\lambda^{-1}\left[P_{\mathrm{bwd}}\left(n-1\right)-\phi_{\mathrm{bwd}}\left(n\right)k_{\mathrm{bwd}}\left(n\right)x_{\mathrm{bwd}}^{H}\left(n\right)P_{\mathrm{bwd}}\left(n-1\right)\right].$$

After the forward and backward PRMCC updates are completed, Bi-PRMCC fuses the estimation results of the two branches through weighted combination to obtain the final channel estimate:

(25) $$\hat{h}\left(n\right)=\eta_{\mathrm{fwd}}{\hat{h}}_{\mathrm{fwd}}\left(n\right)+\eta_{\mathrm{bwd}}{\hat{h}}_{\mathrm{bwd}}\left(n\right),$$

where $\eta_{\mathrm{fwd}}$ and $\eta_{\mathrm{bwd}}$ denote the fusion weights of the forward and backward branches, respectively. To ensure normalization of the fusion weights, the following normalization is adopted:

(26) $$\eta_{\mathrm{fwd}}\leftarrow \frac{\eta_{\mathrm{fwd}}}{\eta_{\mathrm{fwd}}+\eta_{\mathrm{bwd}}},$$

(27) $$\eta_{\mathrm{bwd}}\leftarrow \frac{\eta_{\mathrm{bwd}}}{\eta_{\mathrm{fwd}}+\eta_{\mathrm{bwd}}}.$$

In the experiments of this paper, both $\eta_{\mathrm{fwd}}$ and $\eta_{\mathrm{bwd}}$ are set to 0.5, indicating that the forward and backward branches make equal contributions to the final estimation result.

After fusion, a feedback update mechanism is adopted so that the two branches share a unified channel estimation result in the next iteration:

(28) $${\hat{h}}_{\mathrm{fwd}}\left(n\right)=\hat{h}\left(n\right),$$

(29) $${\hat{h}}_{\mathrm{bwd}}\left(n\right)=\hat{h}\left(n\right).$$

Through this feedback mechanism, the forward and backward branches use the fused estimate as the new initial state after each iteration, thereby preventing long-term divergence between the estimates obtained from the two directions and improving the stability of the bidirectional recursive process.

Under static channel conditions, the backward branch can perform reverse recursion over the entire data length. However, in channel tracking experiments, the true channel may undergo an abrupt change at a certain time instant. If the backward branch directly uses data across the abrupt-change point, different channel states before and after the change may be mixed within the same backward recursive process, thereby degrading the tracking performance.

To address this issue, a segmented reverse-indexing strategy is adopted in Bi-PRMCC. In the channel tracking experiment, the reverse recursion is restricted within each channel-state segment. For the simulated abrupt-change case, the segment boundary is set according to the known change instant, so that the backward branch does not use samples belonging to different channel states. In practical block-based receivers, the segment length can be determined by the training frame length, the expected channel coherence interval, or an external channel-change detector. A shorter segment reduces processing delay and improves responsiveness to channel variations, whereas a longer segment provides more samples for bidirectional estimation. Therefore, the segment length should be selected as a trade-off between estimation accuracy and processing latency.

### 3.3. Pseudocode of the Proposed Bi-PRMCC Algorithm

To provide a clearer description of the implementation procedure, the complete pseudocode of the proposed Bi-PRMCC algorithm is summarized in Algorithm 1. The algorithm consists of a forward PRMCC recursion, a backward PRMCC recursion, and a fusion-feedback operation. In each iteration, the forward branch updates the channel estimate according to the original data order, while the backward branch performs recursive updating according to the reverse index within the current channel segment. The two estimates are then fused to generate the final channel estimate.

Algorithm 1 shows that the proposed Bi-PRMCC algorithm performs two PRMCC recursions in each iteration and then fuses the forward and backward estimates. The maximum correntropy weighting factors suppress the influence of large abnormal errors, while the proportionate matrices enhance the update of dominant taps in sparse channels. Moreover, the segmented reverse-indexing strategy prevents the backward branch from crossing abrupt channel-change points, which improves the tracking stability of the algorithm in time-varying underwater acoustic channels.
**Algorithm 1** The Proposed Bi-PRMCC Algorithm**Input:** ${\left\{x\left(n\right),d\left(n\right)\right\}}_{n=1}^{N}$, parameter set $P=\{L,\lambda ,\delta ,\sigma ,\alpha ,\theta ,\varepsilon ,\eta_{\mathrm{fwd}},\eta_{\mathrm{bwd}}\}$, and segment set S; **Initialization:** ${\hat{h}}_{\mathrm{fwd}}\left(0\right)={\hat{h}}_{\mathrm{bwd}}\left(0\right)=0_{L}$, $P_{\mathrm{fwd}}\left(0\right)=P_{\mathrm{bwd}}\left(0\right)=\delta I_{L}$ **Iteration:** For each channel segment [a,b]∈S, the forward and backward branches are updated using the samples selected according to the original and reverse data orders within the current segment, respectively. For q∈{fwd,bwd}, the PRMCC update is performed as follows:$$e_{q}\left(n\right)=d_{q}\left(n\right)-{\hat{h}}_{q}^{H}\left(n-1\right)x_{q}\left(n\right),\;\phi_{q}\left(n\right)=\exp\left(-\frac{|e_{q}{\left(n\right)|}^{2}}{2\sigma^{2}}\right).$$kq(n)=Pq(n−1)xq(n)λ+ϕq(n)Re{xqH(n)Pq(n−1)xq(n)}.$$g_{q,l}\left(n-1\right)=\theta \left[\frac{1-\alpha }{2L}+\frac{\left(1+\alpha \right)|{\hat{h}}_{q,l}\left(n-1\right)|}{2∥{\hat{h}}_{q}{\left(n-1\right)∥}_{1}+\varepsilon }\right],\;l=1,\ldots ,L.$$$$G_{q}\left(n-1\right)=\mathrm{diag}\left\{g_{q,1}\left(n-1\right),g_{q,2}\left(n-1\right),\ldots ,g_{q,L}\left(n-1\right)\right\}.$$$${\hat{h}}_{q}\left(n\right)={\hat{h}}_{q}\left(n-1\right)+G_{q}\left(n-1\right)k_{q}\left(n\right)\phi_{q}\left(n\right)e_{q}^{*}\left(n\right).$$$$P_{q}\left(n\right)=\lambda^{-1}\left[P_{q}\left(n-1\right)-\phi_{q}\left(n\right)k_{q}\left(n\right)x_{q}^{H}\left(n\right)P_{q}\left(n-1\right)\right].$$
The forward and backward channel estimates are fused according to (25).
The fused estimate is then fed back to the two recursive branches:${\hat{h}}_{\mathrm{fwd}}\left(n\right)=\hat{h}\left(n\right),\;{\hat{h}}_{\mathrm{bwd}}\left(n\right)=\hat{h}\left(n\right).$
**Return:** $\hat{h}$.


### 3.4. Computational Complexity Analysis

To further clarify the implementation cost of the proposed Bi-PRMCC algorithm, the computational complexities of RLS, Bi-RLS, PRLS, RMCC, PRMCC, and Bi-PRMCC are compared. Let *L* denote the channel length. The complexity is mainly evaluated in terms of the number of multiplications required per iteration. Since these recursive algorithms all involve updating the inverse correlation matrix P(n), the dominant computational burden arises from matrix–vector multiplications and matrix-recursion operations. Therefore, the leading-order complexity of all considered methods is associated with $L^{2}$.

As shown in Table 1, RLS, PRLS, RMCC, and PRMCC are all based on a unidirectional recursive structure, and their dominant complexity is $O(L^{2})$. Compared with RLS, PRLS introduces the computation of a proportionate matrix, leading to a slight increase in the linear term. RMCC incorporates the maximum correntropy criterion kernel to reduce the influence of large-error samples on the update process. PRMCC combines both proportionate updating and maximum-correntropy-based error weighting, resulting in a slightly higher cost than PRLS and RMCC.

Bi-RLS and Bi-PRMCC employ a bidirectional iterative structure, in which forward and reverse recursive branches are executed separately. Consequently, their main computational cost is approximately twice that of the corresponding unidirectional algorithms. For the proposed Bi-PRMCC algorithm, each iteration includes one forward PRMCC update and one reverse PRMCC update. The estimates obtained from the two branches are then fused through weighted combination and fed back to both filters. Since the fusion and feedback operations only require vector weighting and assignment, their complexity is merely O(L).

In summary, the proposed Bi-PRMCC algorithm incurs a higher computational cost than the unidirectional PRMCC method, mainly due to the additional bidirectional recursive structure. Nevertheless, its overall complexity remains at the order of $O(L^{2})$, because the dominant cost still comes from inverse correlation matrix updates. Considering its improved performance in non-Gaussian noise suppression, steady-state NMSD reduction, and channel abrupt-change tracking, this additional computational burden is acceptable. Therefore, Bi-PRMCC provides a favorable tradeoff between estimation accuracy and computational complexity for sparse underwater acoustic channel estimation.

## 4. Simulations and Discussion

### 4.1. Simulation Parameter Settings

In the simulations, the tap length of the sparse underwater acoustic channel is set to 64, and a 1500-symbol training sequence is used. To ensure statistical reliability, each performance curve is averaged over 500 independent Monte Carlo trials. Since practical underwater acoustic communication is often affected by impulsive disturbances from ship activity, marine organisms, mechanical vibration, and burst interference, Cauchy-distributed non-Gaussian noise is adopted to model this environment. Its heavy-tailed property captures large-amplitude outliers effectively. The location and scale parameters are set to 0 and 0.2, respectively.

To ensure a fair comparison among different algorithms, the key parameters of each algorithm are uniformly configured and independently tuned. Specifically, the initialization parameter, forgetting factor, kernel width parameter, proportionate control parameters, and bidirectional fusion weights are selected under the same channel model and noise conditions. During parameter tuning, the final parameter configuration of each algorithm is determined by aiming at favorable steady-state NMSD performance while ensuring stable convergence.

In addition, to further investigate the influence of key parameters on algorithm performance, parameter sensitivity experiments are conducted in the following sections, with emphasis on the effects of the kernel width parameter, proportionate control parameters, and forgetting factor on convergence speed, steady-state error, and tracking performance.

To improve the transparency and reproducibility of the experiments, the main parameter settings of all comparison algorithms are provided. The algorithms involved in the comparison include conventional RLS, bidirectional RLS (Bi-RLS), proportionate RLS (PRLS), RMCC, PRMCC, and the proposed Bi-PRMCC algorithm. The specific parameter settings are listed in Table 2.

In Table 2, δ denotes the initialization parameter, λ denotes the forgetting factor, σ denotes the kernel width parameter in the maximum correntropy criterion, α and θ are the proportionate control parameters in the proportionate update mechanism, and $\eta_{\mathrm{fwd}}$ and $\eta_{\mathrm{bwd}}$ represent the forward and backward fusion weights in the bidirectional filtering structure, respectively. For algorithms that do not involve the corresponding mechanism, the associated parameters are denoted by “–”.

### 4.2. Simulated Channel Generation

The experimental evaluation employs shallow-water acoustic channels generated by the Bellhop ray-tracing engine, with the parameter configurations listed in Table 3. By varying the propagation range, two representative underwater acoustic scenarios are constructed: a sparse short-range channel and a long-range channel with severe multipath interference. This setup enables a systematic assessment of the performance gains and robustness of the proposed adaptive filtering algorithms across different underwater acoustic environments.

**Figure 2:**
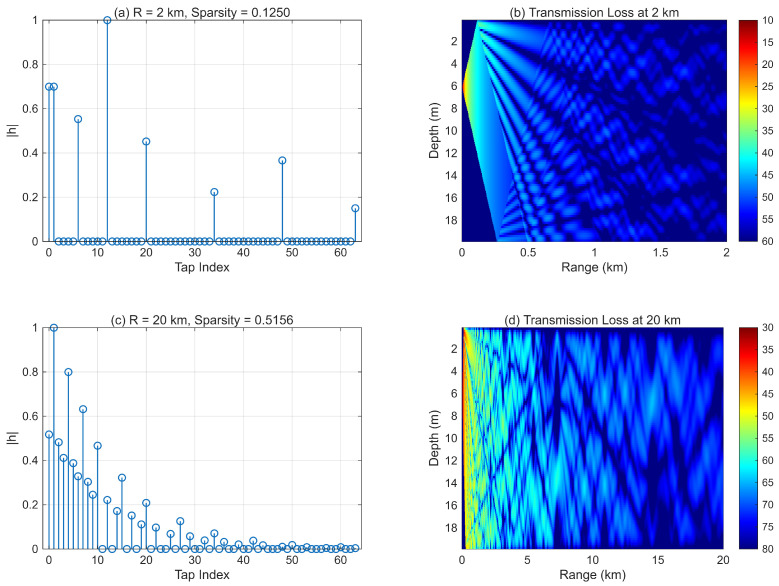
Channel impulse responses and transmission loss profiles under different propagation ranges.

The resulting discrete channel impulse response (CIR) tap profiles, together with the color-coded two-dimensional transmission loss (TL) distributions for both cases, are visualized in Figure 2.

As shown in Figure 2a–d, the underwater acoustic channels exhibit markedly different multipath structures under different propagation ranges. In the short-range scenario (R=2 km), the channel impulse response contains only a few dominant taps, with the energy concentrated along several discrete propagation paths. The sparsity level is 0.1250, indicating a highly sparse channel structure. The corresponding two-dimensional transmission loss (TL) distribution shows that the acoustic energy is mainly confined to the near-range region, leading to a relatively simple multipath interference pattern.

In contrast, the long-range scenario (R=20 km) exhibits denser multipath arrivals and a broader delay spread, with the sparsity level increasing to 0.5156, reflecting a substantial increase in channel complexity. The corresponding TL distribution presents more intricate spatial interference patterns and propagation-path variations, further revealing the stronger multipath effects and transmission loss characteristics associated with long-range underwater acoustic propagation. These two representative channel scenarios provide a physically grounded simulation testbed for evaluating the convergence behavior and steady-state accuracy of the proposed Bi-PRMCC algorithm under different sparsity levels and channel complexities.

### 4.3. Running Time Comparison

**Figure 3:**
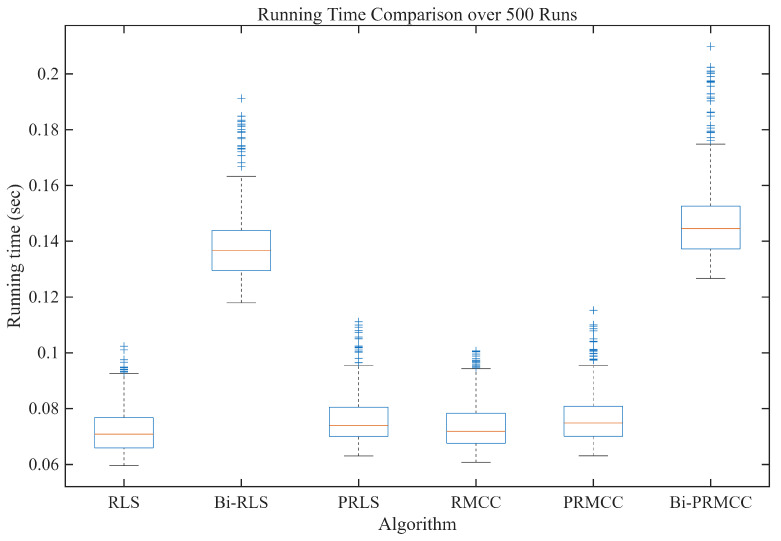
Running time comparison of different algorithms over 500 runs.

To evaluate the computational cost of different algorithms, the running time of RLS, Bi-RLS, PRLS, RMCC, PRMCC, and the proposed Bi-PRMCC was compared under identical simulation conditions. All running time tests were conducted in MATLAB R2025b on a computing platform equipped with an Intel Core i7-14700 CPU (Intel, Santa Clara, CA, USA). A 2-km underwater acoustic channel model was used in the experiment, and its impulse response was normalized before simulation. The number of iterations was set to 1500, and the SNR was set to 20 dB. The same input signal, channel response, and noise sequence were used for all algorithms, and the algorithm parameters were kept consistent with those in the previous experiments. To reduce the influence of MATLAB JIT compilation and fixed execution order, each algorithm was first executed for 5 warm-up runs and then timed over 500 repeated runs with randomly shuffled execution order. The resulting running time distributions are shown in Figure 3.

As shown in Figure 3, RLS has the lowest average running time of 0.072223 s due to its basic recursive structure. PRLS, RMCC, and PRMCC show slightly higher but comparable running times, with average values of 0.076368 s, 0.073572 s, and 0.076603 s, respectively. Bi-RLS and Bi-PRMCC require more computation because of the bidirectional filtering structure, with average running times of 0.138530 s and 0.147340 s, respectively. Although the proposed Bi-PRMCC has the highest computational cost, the increase is reasonable considering its improved steady-state accuracy, robustness against impulsive noise, and block-based channel tracking capability demonstrated in the previous experiments.

### 4.4. Convergence and Steady-State Performance Analysis Under Different Non-Gaussian Noise Conditions

To further verify the robustness and channel estimation capability of the proposed Bi-PRMCC algorithm in complex non-Gaussian noise environments, comparative experiments are conducted under three typical non-Gaussian noise conditions, namely Cauchy noise, α-stable distribution noise, and Middleton noise. The RLS, Bi-RLS, PRLS, RMCC, PRMCC, and Bi-PRMCC algorithms are evaluated. The normalized mean square deviation (NMSD) is adopted as the performance metric and is defined as

(30) $$\mathrm{NMSD}\left(n\right)=10\log_{10}\left(\frac{{∥h_{0}-\hat{h}\left(n\right)∥}_{2}^{2}}{{∥h_{0}∥}_{2}^{2}}\right).$$

where $h_{0}$ denotes the true channel vector, $\hat{h}\left(n\right)$ denotes the estimated channel vector obtained at the *n*th iteration, and ${∥\cdot ∥}_{2}$ represents the Euclidean norm. A lower NMSD indicates a smaller deviation between the estimated channel and the true channel, corresponding to higher channel estimation accuracy and better steady-state performance.

**Figure 4:**
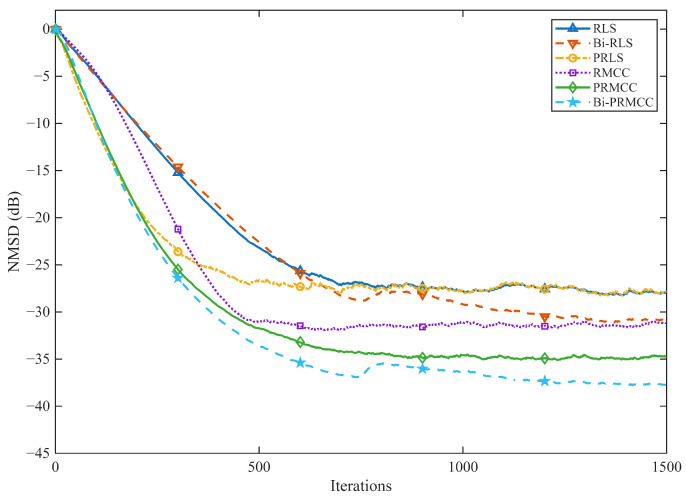
NMSD convergence curves of different algorithms under Cauchy noise.

Under Cauchy noise, the NMSD convergence curves of different algorithms are shown in Figure 4. The steady-state NMSD values of RLS, Bi-RLS, PRLS, RMCC, PRMCC, and Bi-PRMCC are −27.806 dB, −30.768 dB, −27.774 dB, −31.376 dB, −34.869 dB, and −37.587 dB, respectively. Conventional RLS and PRLS exhibit relatively high steady-state errors due to their sensitivity to impulsive noise, whereas RMCC and PRMCC achieve improved performance by introducing the maximum correntropy criterion and the proportionate update mechanism. Among all algorithms, Bi-PRMCC obtains the lowest steady-state NMSD, improving over PRMCC and RMCC by approximately 2.718 dB and 6.211 dB, respectively. This demonstrates that the bidirectional structure further enhances the steady-state estimation accuracy under Cauchy impulsive noise.

**Figure 5:**
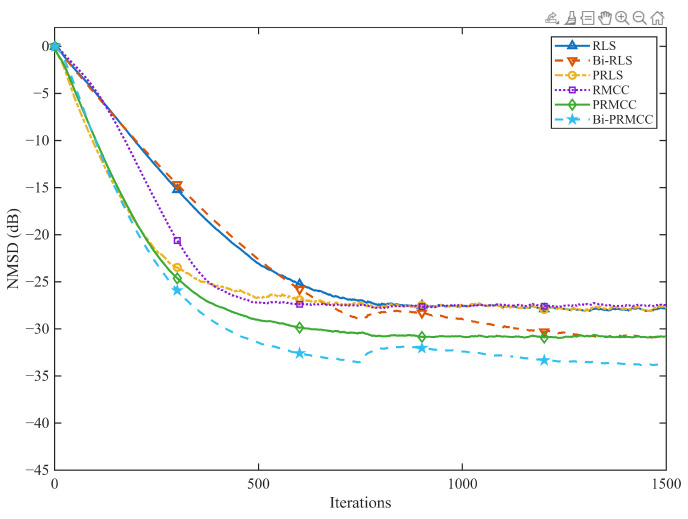
NMSD convergence curves of different algorithms under α-stable distribution noise.

Under α-stable distribution noise, the NMSD convergence curves of different algorithms are shown in Figure 5. The steady-state NMSD values of RLS, Bi-RLS, PRLS, RMCC, PRMCC, and Bi-PRMCC are −27.907 dB, −30.683 dB, −27.840 dB, −27.497 dB, −30.831 dB, and −33.603 dB, respectively. RLS and PRLS exhibit relatively high steady-state errors, indicating the limited robustness of conventional second-order-error-based algorithms under strong non-Gaussian noise. PRMCC effectively reduces the steady-state error by combining the maximum correntropy criterion with the proportionate update mechanism. Furthermore, Bi-PRMCC jointly exploits forward and backward information and achieves the lowest steady-state NMSD, improving over PRMCC by approximately 2.772 dB, which demonstrates stronger resistance to impulsive interference.

**Figure 6:**
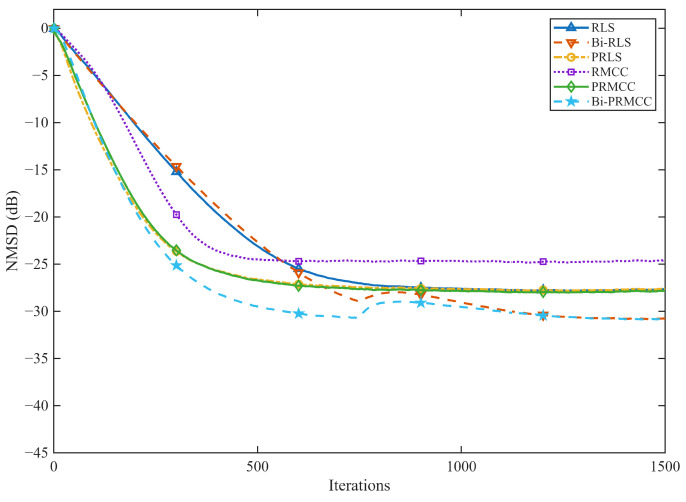
NMSD convergence curves of different algorithms under Middleton noise.

Under Middleton noise, the NMSD convergence curves of different algorithms are shown in Figure 6. The steady-state NMSD values of RLS, Bi-RLS, PRLS, RMCC, PRMCC, and Bi-PRMCC are −27.752 dB, −30.695 dB, −27.724 dB, −24.710 dB, −27.930 dB, and −30.728 dB, respectively. Unlike the previous two noise types, RMCC exhibits a relatively high steady-state error under Middleton noise, indicating that the maximum correntropy criterion alone may have certain parameter sensitivity. Both Bi-RLS and Bi-PRMCC achieve favorable performance, suggesting that the bidirectional filtering structure helps improve estimation accuracy. Among all algorithms, Bi-PRMCC obtains the lowest steady-state NMSD, slightly outperforming Bi-RLS and demonstrating its stability under Middleton noise.

Overall, the experimental results under the three non-Gaussian noise conditions show that the proposed Bi-PRMCC algorithm achieves the best steady-state NMSD performance under Cauchy noise, α-stable distribution noise, and Middleton noise. In particular, under strong impulsive noise environments such as Cauchy noise and α-stable distribution noise, Bi-PRMCC exhibits obvious advantages over conventional RLS-based algorithms, RMCC, and PRMCC.

This demonstrates that the maximum correntropy criterion can effectively reduce the influence of non-Gaussian impulsive noise on the error update process, the proportionate update mechanism can improve the estimation accuracy of sparse channels, and the bidirectional filtering structure can further enhance the convergence stability and steady-state performance of the algorithm. Therefore, Bi-PRMCC is more suitable for sparse underwater acoustic channel estimation tasks under complex non-Gaussian noise backgrounds.

### 4.5. Reconvergence Analysis Under Abrupt Channel Changes

**Figure 7:**
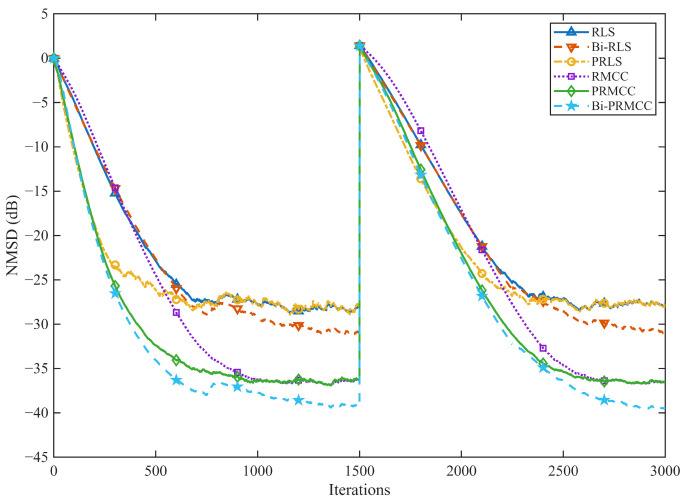
NMSD tracking curves of different algorithms under abrupt channel changes.

To further evaluate the reconvergence capability of the proposed Bi-PRMCC algorithm under block-based channel tracking conditions, an abrupt channel-change experiment is conducted. The total number of iterations is set to 3000, and an abrupt channel change is introduced at the 1500th iteration to simulate rapid channel variations caused by platform motion, ocean environmental changes, or propagation path variations in practical underwater acoustic communication. The NMSD tracking curves of different algorithms in this scenario are shown in Figure 7.

As shown in Figure 7, all algorithms gradually converge during the first 1500 iterations, but their steady-state NMSD levels differ noticeably. Conventional RLS and PRLS retain basic adaptive updating capability; however, their steady-state errors remain relatively high under complex noise and sparse underwater acoustic channel conditions. Bi-RLS achieves a lower NMSD by introducing bidirectional filtering. In contrast, RMCC and PRMCC provide better steady-state accuracy because the maximum correntropy criterion suppresses abnormal error samples. PRMCC further improves sparse-channel estimation through proportionate tap updating.

When an abrupt channel change occurs at the 1500th iteration, the NMSD curves of all algorithms rise sharply. This indicates that the previous filter weights no longer match the new channel state and that the algorithms must reconverge. RLS and PRLS show large error increases and still maintain relatively high NMSD levels after reconvergence, revealing limited tracking capability. Bi-RLS recovers faster due to its bidirectional structure. Nevertheless, since Bi-RLS is based on the second-order error criterion, its robustness against non-Gaussian impulsive interference remains limited.

Compared with the benchmark algorithms, the proposed Bi-PRMCC exhibits superior reconvergence performance after the abrupt channel variation under the block-based processing setting. Although its NMSD increases temporarily after the change, it rapidly reconverges and reaches a lower steady-state error level. This demonstrates that Bi-PRMCC can effectively readapt the filter weights to the new channel state within the considered segment-wise estimation framework. Its advantage is mainly attributed to three factors: maximum correntropy weighting improves robustness to abnormal samples, proportionate updating enhances dominant-tap estimation, and bidirectional filtering exploits complementary forward and backward information for faster recovery.

In summary, for the tracking experiment with 3000 iterations and an abrupt channel change at the 1500th iteration, Bi-PRMCC achieves a lower steady-state error before the channel change and rapidly recovers after the variation. Compared with RLS, Bi-RLS, PRLS, RMCC, and PRMCC, the proposed algorithm shows better overall performance in steady-state estimation accuracy, abrupt-change response capability, and block-based reconvergence stability. These results indicate that Bi-PRMCC is effective for block-based adaptive channel estimation under abrupt channel variations.

### 4.6. Steady-State NMSD Performance Analysis Under Different SNR Conditions

**Figure 8:**
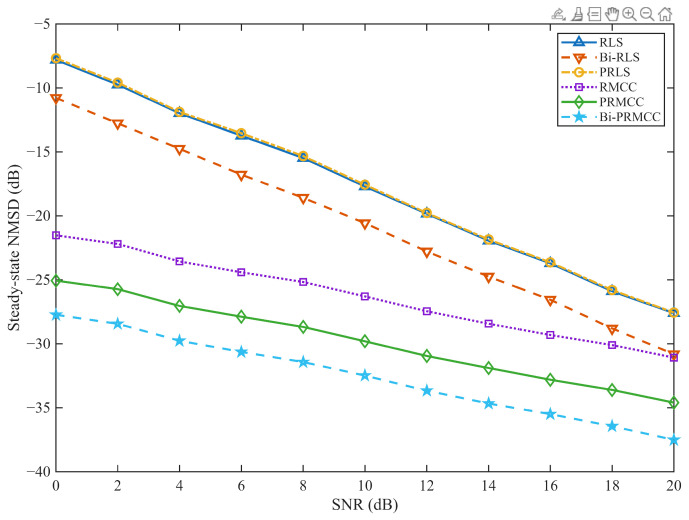
Steady-state NMSD curves of different algorithms versus SNR.

To further evaluate the steady-state channel estimation performance of different algorithms under different signal-to-noise ratio (SNR) conditions, this paper presents the steady-state NMSD results of RLS, Bi-RLS, PRLS, RMCC, PRMCC, and the proposed Bi-PRMCC algorithm when the SNR varies from 0 dB to 20 dB. The steady-state NMSD curves of different algorithms versus SNR are shown in Figure 8.

As shown in Figure 8, the steady-state NMSD values of all algorithms gradually decrease as the SNR increases, indicating that the channel estimation accuracy of each algorithm improves when the noise intensity decreases. However, the performance differences among different algorithms remain significant. Over the entire SNR range, the steady-state NMSD values of conventional RLS and PRLS remain relatively high, and their performance is close. For example, when the SNR is 0 dB, the steady-state NMSD values of RLS and PRLS are −7.8091 dB and −7.6786 dB, respectively. When the SNR increases to 20 dB, they decrease to −27.6060 dB and −27.5590 dB, respectively. This indicates that although RLS-based algorithms can improve estimation accuracy as the SNR increases, their robustness remains limited under low-SNR and complex noise conditions.

Bi-RLS shows a significant improvement over conventional RLS. Under all SNR conditions, the steady-state NMSD of Bi-RLS is lower than that of RLS. For example, when the SNR is 0 dB, the steady-state NMSD of Bi-RLS is −10.779 dB, which is approximately 2.970 dB lower than that of RLS. When the SNR is 20 dB, Bi-RLS reaches −30.815 dB, which is approximately 3.209 dB lower than RLS. This indicates that the bidirectional filtering structure can effectively exploit the information from forward and backward data, thereby improving steady-state estimation accuracy.

For algorithms based on the maximum correntropy criterion, RMCC, PRMCC, and Bi-PRMCC can still maintain relatively low steady-state NMSD values under low-SNR conditions, showing stronger noise suppression capability. When the SNR is 0 dB, the steady-state NMSD values of RMCC, PRMCC, and Bi-PRMCC are −21.519 dB, −25.060 dB, and −27.733 dB, respectively, which are significantly better than those of RLS-based algorithms. This indicates that the maximum correntropy criterion can effectively suppress the influence of noise and abnormal error samples on weight updates, thereby improving the robustness of the algorithm in low-SNR environments.

A further comparison between PRMCC and RMCC shows that PRMCC outperforms RMCC under all SNR conditions. For example, when the SNR is 10 dB, the steady-state NMSD of RMCC is −26.295 dB, whereas that of PRMCC decreases to −29.803 dB. When the SNR is 20 dB, RMCC obtains −31.083 dB, while PRMCC reaches −34.597 dB. This demonstrates that the proportionate update mechanism can better exploit the structural characteristics of sparse underwater acoustic channels and improve the estimation accuracy of dominant taps, thereby achieving lower steady-state errors.

The proposed Bi-PRMCC algorithm achieves the best steady-state NMSD performance over the entire SNR range. When the SNR is 0 dB, the steady-state NMSD of Bi-PRMCC is −27.733 dB, which further reduces the NMSD by approximately 2.673 dB compared with PRMCC. When the SNR is 20 dB, Bi-PRMCC reaches −37.511 dB, which is approximately 2.914 dB lower than PRMCC. These results indicate that Bi-PRMCC not only has stronger noise suppression capability under low-SNR conditions but also maintains higher steady-state estimation accuracy under high-SNR conditions.

Overall, as the SNR increases from 0 dB to 20 dB, the steady-state NMSD of Bi-PRMCC decreases from −27.733 dB to −37.511 dB and remains consistently better than those of the other comparison algorithms. This shows that the combination of the maximum correntropy criterion, proportionate update mechanism, and bidirectional filtering structure can effectively improve the steady-state performance of the algorithm under different SNR conditions. Compared with RLS, Bi-RLS, PRLS, RMCC, and PRMCC, the proposed Bi-PRMCC exhibits better robustness and channel estimation accuracy in both low-SNR and high-SNR scenarios.

### 4.7. Ablation Study of the Bidirectional Structure

To further verify the contribution of the bidirectional filtering structure to the performance improvement of the proposed Bi-PRMCC algorithm, an ablation study is conducted. PRMCC is selected as the baseline algorithm and compared with Bi-PRMCC after introducing the bidirectional structure. Both algorithms adopt the same maximum correntropy criterion and proportionate update mechanism, while Bi-PRMCC further incorporates a joint forward and backward estimation structure. Therefore, this experiment can directly evaluate the influence of the bidirectional structure on steady-state channel estimation performance.

**Figure 9:**
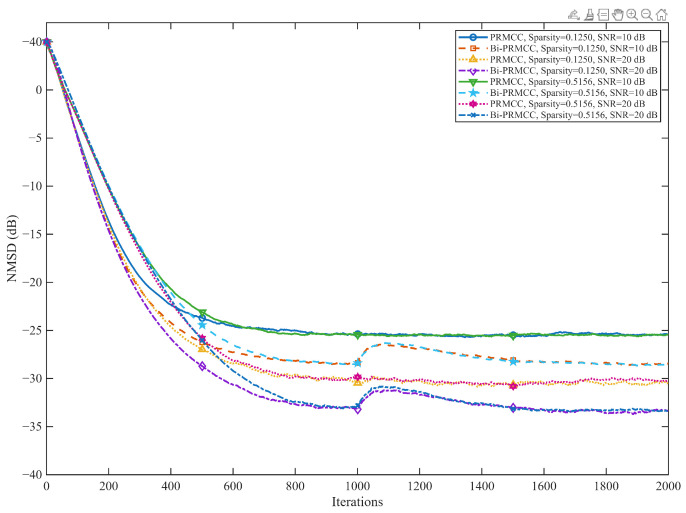
Ablation results of PRMCC and Bi-PRMCC under different sparsity levels and SNR conditions.

The experiments are conducted under two channel sparsity levels and two SNR conditions. The channel with a sparsity level of 0.1250 corresponds to a relatively sparse short-range underwater acoustic channel, while the channel with a sparsity level of 0.5156 corresponds to a long-range underwater acoustic channel with more complex multipath propagation. The SNR values are set to 10 dB and 20 dB. The quantitative steady-state NMSD results are summarized in Table 4, and the corresponding comparison is illustrated in Figure 9.

As shown in Table 4 and Figure 9, Bi-PRMCC achieves lower steady-state NMSD than PRMCC under all tested conditions. For the short-range sparse channel with a sparsity level of 0.1250, Bi-PRMCC obtains performance improvements of 3.037 dB and 2.947 dB at SNR values of 10 dB and 20 dB, respectively, indicating that the bidirectional structure can further enhance the estimation capability for dominant propagation paths in sparse channels.

For the long-range complex multipath channel with a sparsity level of 0.5156, Bi-PRMCC still maintains a stable advantage over PRMCC, achieving improvements of 2.980 dB and 3.083 dB at SNR values of 10 dB and 20 dB, respectively. This indicates that the bidirectional filtering structure remains effective even under denser multipath propagation and higher channel complexity.

Furthermore, the performance gain of Bi-PRMCC over PRMCC remains around 3 dB under different sparsity and SNR conditions, with an average improvement of approximately 3.012 dB. This suggests that the benefit introduced by the bidirectional structure is stable and does not depend on a specific channel sparsity level or SNR condition.

Overall, the ablation results verify that the performance improvement of Bi-PRMCC mainly originates from the introduction of the bidirectional filtering structure. By fusing forward and backward estimation information, the proposed structure further reduces the steady-state NMSD while retaining the non-Gaussian noise suppression capability of the maximum correntropy criterion and the sparse channel estimation advantage of the proportionate update mechanism. These results confirm the effectiveness of the bidirectional structure and further demonstrate the stronger steady-state estimation capability and robustness of the proposed Bi-PRMCC algorithm.

### 4.8. Key Parameter Selection and Sensitivity Analysis

To analyze the influence of key parameters on the convergence performance and steady-state accuracy of the Bi-PRMCC algorithm, this paper investigates the effects of the forgetting factor λ, the kernel width parameter σ, and the proportionate control parameter θ on the NMSD curves. It should be noted that the parameter selection in this paper is not based solely on the lowest steady-state NMSD in a single curve. Instead, the final parameter configuration is determined by comprehensively considering convergence speed, steady-state error, curve stability, and general applicability in subsequent experimental scenarios. The final adopted parameters are σ=1.2, θ=64, and λ=0.995.

**Figure 10:**
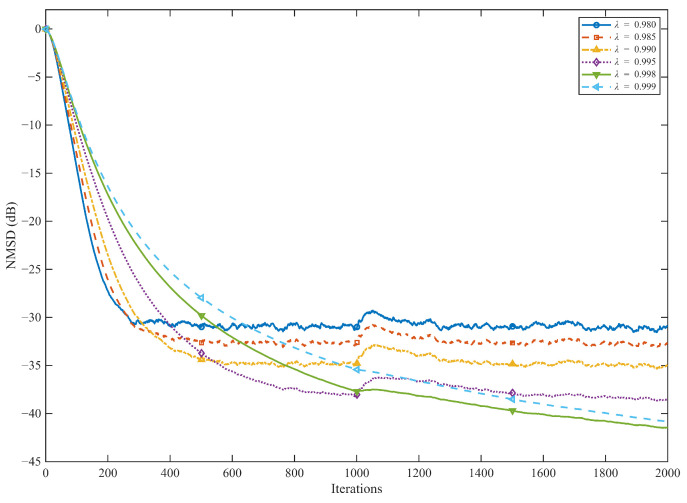
NMSD convergence curves of the Bi-PRMCC algorithm under different values of λ.

The influence of the forgetting factor λ on the convergence behavior of the Bi-PRMCC algorithm is shown in Figure 10.

As shown in Figure 10, the forgetting factor λ has a significant influence on both convergence speed and steady-state error. When λ is relatively small, such as λ=0.980 and λ=0.985, the algorithm decreases rapidly in the initial stage, but its NMSD after convergence remains relatively high, resulting in poor steady-state performance. This is because a smaller forgetting factor reduces the role of historical data in recursive updating. As λ increases, the steady-state NMSD gradually decreases. Although λ=0.998 and λ=0.999 can obtain lower final NMSD values, their early convergence speeds are relatively slow, and an excessively large λ may weaken the response capability to new channel states. In comparison, λ=0.995 achieves a favorable trade-off between convergence speed and steady-state accuracy. Therefore, λ=0.995 is selected as the forgetting factor in this paper.

**Figure 11:**
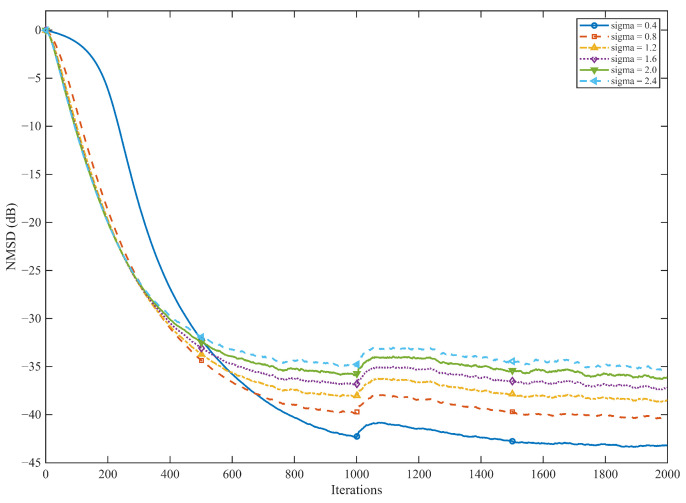
NMSD convergence curves of the Bi-PRMCC algorithm under different values of σ.

The influence of the kernel width parameter σ on the convergence behavior of the Bi-PRMCC algorithm is shown in Figure 11.

As shown in Figure 11, the kernel width σ has a significant influence on the performance of Bi-PRMCC under the maximum correntropy criterion. When σ=0.4, the algorithm eventually achieves the lowest steady-state NMSD, but its early convergence is noticeably slower. This indicates that an excessively small kernel width makes the update process more conservative and may increase the sensitivity of the algorithm to specific noise conditions and error distributions.

As σ increases, the early convergence speed is improved, whereas the steady-state NMSD gradually becomes higher. This suggests that an overly large kernel width weakens the suppression capability against abnormal errors and non-Gaussian impulsive noise. Although σ=1.2 does not yield the lowest final NMSD in this group of curves, it provides stable convergence, relatively low steady-state error, and better general applicability in subsequent experiments. Therefore, σ=1.2 is selected as the kernel width parameter.

**Figure 12:**
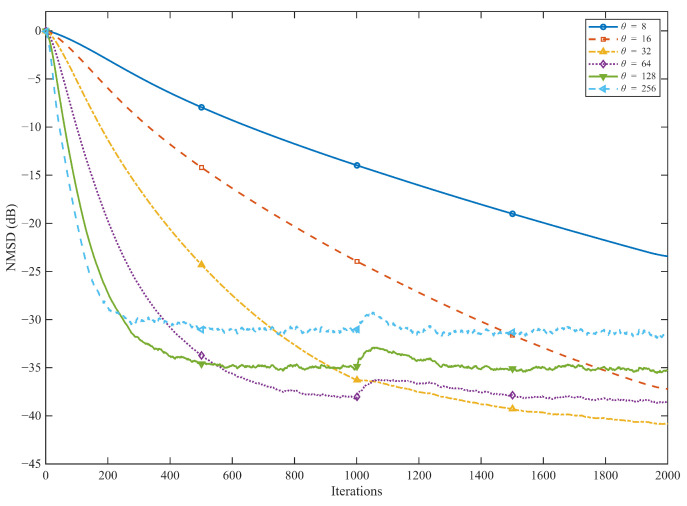
NMSD convergence curves of the Bi-PRMCC algorithm under different values of θ.

The influence of the proportionate control parameter θ on the convergence behavior of the Bi-PRMCC algorithm is shown in Figure 12.

As shown in Figure 12, the proportionate control parameter θ mainly governs the update allocation among channel taps. When θ=8, the convergence speed is slow and the final NMSD remains relatively high, indicating that an excessively small θ cannot sufficiently exploit the proportionate adaptation mechanism. As θ increases to 16 and 32, the estimation performance is noticeably improved, suggesting that a stronger proportionate update helps enhance the adaptation of dominant taps in sparse underwater acoustic channels.

Although θ=32 improves the steady-state behavior, it still requires a relatively long convergence period in the early and middle iteration stages. In contrast, θ=64 achieves faster NMSD reduction at the initial stage and maintains a lower steady-state error, indicating a better balance between convergence speed and estimation accuracy. When θ is further increased to 128 and 256, the algorithm tends to remain at a higher error level in the later stage, implying that an overly large proportionate control parameter may degrade steady-state performance.

Overall, the sensitivity analysis demonstrates that λ, σ, and θ have significant effects on the convergence behavior and steady-state performance of Bi-PRMCC. A larger λ improves steady-state accuracy but may weaken tracking capability, whereas a smaller σ enhances impulsive-noise suppression but may slow early convergence. An appropriate θ strengthens the update of dominant sparse-channel taps, while an excessive value may reduce steady-state accuracy. Therefore, σ=1.2, θ=64, and λ=0.995 are adopted in the subsequent experiments to achieve a balanced trade-off among convergence speed, accuracy, stability, and tracking adaptability.

**Figure 13:**
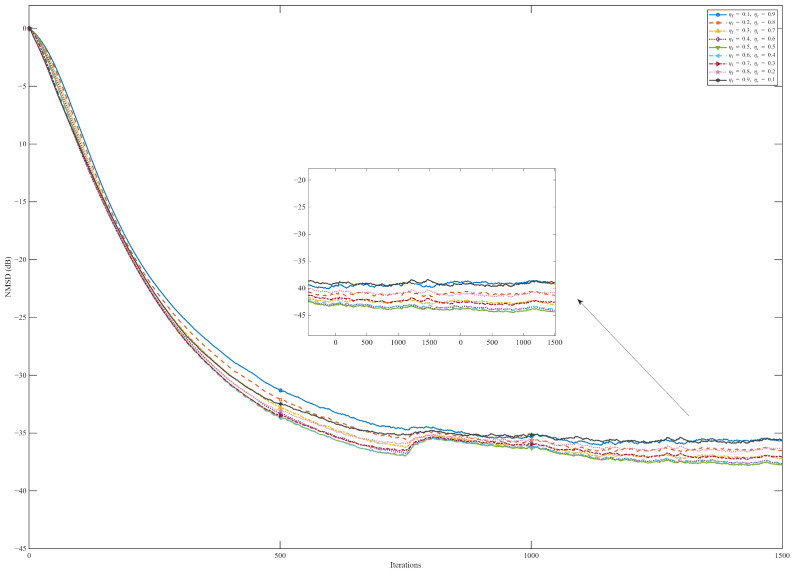
NMSD convergence curves of the Bi-PRMCC algorithm under different fusion weights.

To further analyze the influence of the bidirectional fusion weights on the algorithm performance, a parameter sensitivity analysis is conducted for the forward weight $\eta_{f}$ and the reverse weight $\eta_{r}$. The experiment is carried out using the 2-km underwater acoustic channel, with the SNR set to 20 dB. The parameters are fixed as σ=1.2, θ=64, and λ=0.995, and only the fusion weights are varied. Specifically, $\eta_{f}$ is changed from 0.1 to 0.9, while $\eta_{r}=1-\eta_{f}$ is maintained. The NMSD convergence curves under different fusion weight combinations are shown in Figure 13.

As shown in Figure 13, the fusion weights have a clear influence on the steady-state performance of Bi-PRMCC. When the fusion weight is strongly biased toward either the forward or reverse recursion, such as $\left(\eta_{f},\eta_{r}\right)=\left(0.1,0.9\right)$ or (0.9,0.1), the steady-state NMSD remains relatively high, with values of −35.669 dB and −35.683 dB, respectively. As the forward and reverse weights become more balanced, the steady-state NMSD gradually decreases. When $\eta_{f}=\eta_{r}=0.5$, the algorithm achieves the lowest steady-state NMSD of −37.587 dB. In addition, the results under (0.4,0.6) and (0.6,0.4) are also close, with steady-state NMSD values of −37.441 dB and −37.443 dB, respectively. This indicates that the forward and reverse recursions can provide complementary estimation information, while excessive reliance on a single direction may weaken the performance gain of the bidirectional filtering structure. Therefore, $\eta_{f}=\eta_{r}=0.5$ is adopted in the subsequent experiments.

### 4.9. NMSD Performance Evaluation Using Measured Underwater Acoustic Channel Data

To further evaluate the channel identification performance of the proposed algorithm under practical underwater acoustic conditions, a measured underwater acoustic channel from the IEEE DataPort open-access dataset In-Band Full-Duplex Underwater Acoustic Communication Measurements: Self-interference is used in this experiment [31].

The dataset provides lake-trial underwater acoustic communication measurements and processed channel impulse responses, which can reflect practical propagation effects such as multipath propagation, channel fading, and environmental uncertainties.

**Figure 14:**
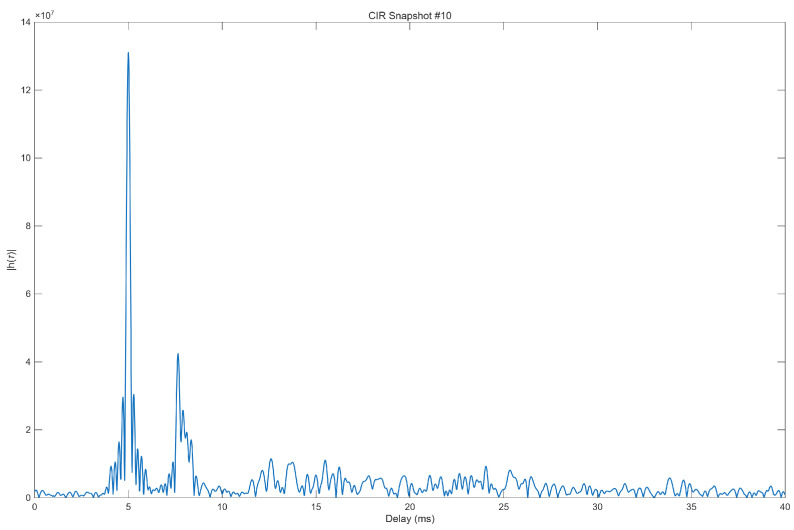
Extracted measured underwater acoustic channel response at a single time instant.

In this experiment, the CIR data file is used as the source of the measured channel. Specifically, one channel impulse response at a selected time instant is extracted from the CIR matrix in CIR@1610#1472.mat and used as the reference channel for identification. The extracted measured channel response is shown in Figure 14. For a fair comparison, all algorithms are tested using the same input signal, measured channel, and noise condition. The RLS, Bi-RLS, PRLS, RMCC, PRMCC, and proposed Bi-PRMCC algorithms are compared under the same measured underwater acoustic channel. The number of iterations is set to 1500, and the signal-to-noise ratio is set to 20 dB.

**Figure 15:**
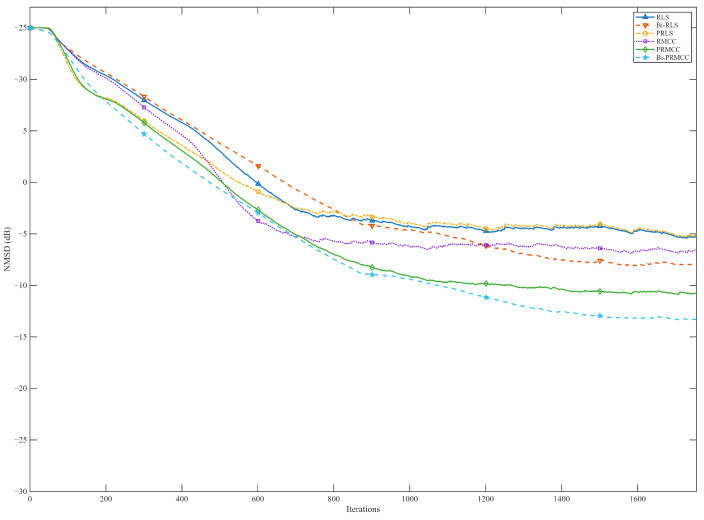
NMSD performance comparison of different algorithms under the measured underwater acoustic channel.

The NMSD comparison results of different algorithms under the measured underwater acoustic channel are shown in Figure 15. It can be observed that the proposed Bi-PRMCC algorithm achieves the lowest steady-state NMSD after convergence, indicating that its estimated channel is closest to the measured true channel. The steady-state NMSD values of RLS, Bi-RLS, PRLS, RMCC, PRMCC, and Bi-PRMCC are −19.723 dB, −22.845 dB, −19.574 dB, −21.555 dB, −25.650 dB, and −28.065 dB, respectively.

From the comparison results, the conventional RLS algorithm can achieve basic channel identification, but its steady-state error is relatively high under the measured underwater acoustic channel. Compared with RLS, Bi-RLS obtains a lower NMSD, which indicates that the bidirectional update mechanism can improve the channel estimation performance. The steady-state NMSD of PRLS is close to that of RLS in this measured channel case, suggesting that the proportional update alone does not bring a significant improvement in this scenario. The RMCC algorithm performs better than RLS and PRLS, which demonstrates the robustness of the maximum correntropy criterion in a noisy environment. By further combining the proportional update with the maximum correntropy criterion, PRMCC reduces the steady-state NMSD to −25.650 dB and outperforms RLS, Bi-RLS, PRLS, and RMCC.

Among all compared algorithms, the proposed Bi-PRMCC algorithm achieves the best performance with a steady-state NMSD of −28.065 dB. Compared with RLS, Bi-PRMCC reduces the NMSD by approximately 8.342 dB. Compared with Bi-RLS, it achieves an improvement of approximately 5.220 dB. In addition, compared with PRMCC, the proposed Bi-PRMCC further reduces the NMSD by approximately 2.415 dB. These results show that the proposed algorithm can effectively combine the advantages of bidirectional update, proportional update, and maximum correntropy criterion, leading to improved channel identification accuracy and robustness under measured underwater acoustic channel conditions.

## 5. Conclusions and Future Work

This paper proposed a bidirectional proportionate recursive maximum correntropy criterion algorithm, termed Bi-PRMCC, for sparse underwater acoustic channel estimation under impulsive noise and time-varying channel conditions. By combining maximum correntropy weighting, proportionate adaptation, and forward–backward filtering, the proposed algorithm can suppress abnormal error samples while enhancing the update of dominant channel taps.

The performance of Bi-PRMCC was evaluated using Bellhop-generated underwater acoustic channels under Cauchy noise, α-stable noise, and Middleton noise. Compared with RLS, Bi-RLS, PRLS, RMCC, and PRMCC, Bi-PRMCC achieved lower steady-state NMSD and more stable convergence in all tested noise environments. The SNR experiments further showed that the proposed algorithm maintains the best estimation accuracy over different noise levels. In the channel tracking experiment with an abrupt channel change, Bi-PRMCC also exhibited faster reconvergence and better block-based channel variation adaptation than the benchmark methods.

An ablation study verified the contribution of the bidirectional structure, where Bi-PRMCC provided about 3 dB improvement over PRMCC under different sparsity and SNR conditions. In addition, the measured underwater acoustic channel experiment based on the IEEE DataPort dataset further confirmed the practical effectiveness of the proposed method. Bi-PRMCC achieved a steady-state NMSD of −28.065 dB, outperforming RLS, Bi-RLS, PRLS, RMCC, and PRMCC.

Overall, the proposed Bi-PRMCC algorithm provides improved robustness, estimation accuracy, and block-based reconvergence capability for sparse underwater acoustic channel estimation. Future work will focus on reducing computational complexity, developing adaptive parameter selection strategies, and extending the method to MIMO underwater acoustic communication and joint channel estimation and equalization.

## Figures and Tables

**Table 1:** Computational complexity comparison of different recursive algorithms.

Algorithm	Complexity per Iteration	Main Parameters
RLS	$O(3L^{2}+4L)$	λ,δ
Bi-RLS	$O(6L^{2}+10L)$	$\lambda ,\delta ,\eta_{\mathrm{fwd}},\eta_{\mathrm{bwd}}$
PRLS	$O(3L^{2}+7L)$	λ,δ,α,θ
RMCC	$O(3L^{2}+5L)$	λ,δ,σ
PRMCC	$O(3L^{2}+8L)$	λ,δ,σ,α,θ
Bi-PRMCC	$O(6L^{2}+19L)$	$\lambda ,\delta ,\sigma ,\alpha ,\theta ,\eta_{\mathrm{fwd}},\eta_{\mathrm{bwd}}$

**Table 2:** Parameter settings of different algorithms.

Algorithm	δ	λ	σ	α	θ	$\eta_{\mathrm{fwd}}$	$\eta_{\mathrm{bwd}}$
RLS	100	0.995	–	–	–	–	–
Bi-RLS	100	0.995	–	–	–	0.5	0.5
PRLS	100	0.995	–	−0.5	64	–	–
RMCC	100	0.990	1.2	–	–	–	–
PRMCC	100	0.995	1.2	−0.5	64	–	–
Bi-PRMCC	100	0.995	1.2	−0.5	64	0.5	0.5

**Table 3:** Configuration of environmental parameters for the simulated underwater acoustic channels.

Physical Parameter	Case I (Short-Range)	Case II (Long-Range)
Water depth (m)	20	20
Speed of sound (m/s)	1548.52	1548.52
Center frequency (kHz)	21	21
Transmitter depth (m)	6	6
Receiver depth (m)	6	6
Horizontal range *R* (km)	2	20
Grazing-angle span α (°)	$\left[\pm 3^{\circ }\right]$	$\left[\pm 3^{\circ }\right]$

**Table 4:** Numerical ablation results of PRMCC and Bi-PRMCC under different sparsity levels and SNR conditions.

Sparsity	SNR	PRMCC NMSD	Bi-PRMCC NMSD	Improvement
0.1250	10 dB	−30.372 dB	−33.409 dB	3.037 dB
0.1250	20 dB	−35.482 dB	−38.429 dB	2.947 dB
0.5156	10 dB	−30.490 dB	−33.470 dB	2.980 dB
0.5156	20 dB	−35.201 dB	−38.284 dB	3.083 dB

## Data Availability

The original contributions presented in this study are included in the article. Further inquiries can be directed to the corresponding author.
